# Efficacy outcomes between tarlatamab and real-world physicians’ choice of therapies for previously treated extensive stage small cell lung cancer

**DOI:** 10.1093/oncolo/oyaf256

**Published:** 2025-08-21

**Authors:** Jessie Wang, Gautam Sajeev, Xinglei Chai, Rumbidzai Takundwa, Franziska Dirnberger, Xerxes Pundole, Malaika Pastel, Hongbo Yang, Umit Tapan

**Affiliations:** Amgen Inc., Thousand Oaks, CA 91320, United States; Analysis Group, Inc., Boston, MA 02199, United States; Analysis Group, Inc., Boston, MA 02199, United States; Amgen Ltd, Uxbridge UB8 1DH, United Kingdom; Amgen GmbH, Munich 80992, Germany; Amgen Inc., Thousand Oaks, CA 91320, United States; Amgen Inc., Thousand Oaks, CA 91320, United States; Analysis Group, Inc., Boston, MA 02199, United States; Section of Hematology & Medical Oncology, Boston University Chobanian & Avedisian School of Medicine, and Boston Medical Center, Boston, MA 02118, United States

**Keywords:** external control arm, indirect treatment comparison, small cell lung cancer, tarlatamab, real-world

## Abstract

**Background:**

Tarlatamab, a bispecific T-cell engager immunotherapy, showed durable response with promising survival outcomes in patients with previously treated small cell lung cancer (SCLC) in the phase 2 DeLLphi-301 study. Given the lack of a comparator in DeLLphi-301, this analysis aimed to evaluate the relative efficacy of tarlatamab against physicians’ choice of therapies in real-world practice.

**Patients and Methods:**

This analysis compared the outcomes of patients in DeLLphi-301 who received tarlatamab 10 mg (*n* = 97) with patients in real-world cancer clinics captured in the Flatiron Health database who received third or later-line comparator therapies for SCLC (*n* = 184). Propensity score weighting was used to adjust for differences in key prognostic factors between cohorts. Overall survival (OS), progression-free survival (PFS), time to treatment discontinuation (TTD), time to next treatment or death (TTNTD), and objective response rate (ORR) were compared after weighting.

**Results:**

Tarlatamab was associated with significantly longer OS, PFS, TTD, and TTNTD, and higher ORR vs comparator therapies. After weighting, the hazard ratios (95% confidence interval [CI]) of tarlatamab vs comparator therapies were 0.45 (0.30, 0.68) for OS, 0.61 (0.43, 0.90) for PFS, 0.57 (0.39, 0.84) for TTD, and 0.45 (0.30, 0.66) for TTNTD. The odds ratio for ORR was 2.80 (95% CI, 1.44, 5.83).

**Conclusion:**

The study findings suggest that tarlatamab offers potential clinical benefits relative to comparator treatments. This analysis underscores the potential of tarlatamab to become a new therapeutic option for previously treated SCLC, a disease that has historically been associated with extremely poor outcomes and limited treatment options.

Implications for Practice The DeLLphi-301 trial of tarlatamab demonstrated promising survival among patients with extensive stage small-cell lung cancer (ES-SCLC), but it lacked a direct comparator. The findings of this indirect comparison study suggest that tarlatamab, a bispecific T-cell engager immunotherapy, offers a higher response rate and significant survival benefits compared to previously available real-world therapies for ES-SCLC. The results can enhance patient care, helping to inform treatment decision-making for patients with ES-SCLC who have not responded to or relapsed after prior platinum-based chemotherapy. In clinical practice, tarlatamab could become a preferred treatment option, prompting potential changes to treatment guidelines for ES-SCLC.

## Introduction

Small cell lung cancer (SCLC) is a high-grade neuroendocrine tumor marked by an exceptionally high proliferative rate, early metastasis, extremely aggressive nature, and a poor prognosis.^1^ Patients often present with highly symptomatic disease and are at risk of rapid deterioration. The disease accounts for approximately 10%-15% of lung cancers in the United States (US)^2^ and, despite high response rates to first-line (1L) platinum-based regimens, the majority of patients rapidly experience disease progression.^3^ After unsuccessful 1L treatment, limited therapeutic options are available. Second-line or later (2L+) treatments mostly consist of various palliative chemotherapies that are highly toxic and offer short-lived activity.^3^^,^^4^ There have been few advancements in the treatment of SCLC in the past decade other than the addition of programmed cell death protein 1 (PD-1) or PD-1 ligand (PD-L1) inhibitors as 1L options.^5^ Thus, there is an urgent need for novel and highly effective therapeutic options for extensive stage SCLC (ES-SCLC) considering that the median overall survival (OS) for relapsed disease is approximately 4.0-5.6 months in the real world, regardless of prior PD-1 or PD-L1 inhibitor use.^6^

Currently, there are no US Food and Drug Administration (FDA) approved therapies specifically indicated for third-line and beyond (3L+) for ES-SCLC. Therapies used in 3L+ settings include chemotherapies such as topotecan, lurbinectedin, taxane-based regimens, rechallenge with platinum-based regimens with or without immunotherapy, or immunotherapies.^7–9^ Each of these therapies has demonstrated limited survival benefits, with median OS ranging from 5.0 to 9.7 months, and are associated with substantial cumulative toxicity.^10–24^

Tarlatamab is a bi-specific T-cell engager molecule immunotherapy with an extended half-life that targets delta-like ligand 3 on SCLC cells and CD3 on T cells, leading to T-cell-mediated cancer cell lysis.^25^ Tarlatamab was evaluated in the pivotal phase 2, open-label DeLLphi-301 trial (NCT05060016^26^) among patients with ES-SCLC whose disease had progressed or recurred following a platinum-based regimen and at least one other line of therapy (LOT).^27^ Tarlatamab demonstrated rapid and durable responses in DeLLphi-301—among 100 patients receiving tarlatamab at the target dose of 10 mg, 40% (97.5% confidence interval [CI]: 29% to 52%) achieved objective response, with a median time to response of 1.4 months and median duration of response of 9.7 (95% CI, 6.9, not estimable [NE]) months.^27^^,^^28^ Furthermore, tumor shrinkage was observed in 72% of patients with a median duration of disease control of 6.9 (95% CI, 5.4-8.6) months. At long-term follow-up, the median progression-free survival (PFS) was 4.3 months (95% CI, 2.9-5.6 months) while the median OS was 15.2 (10.8, NE) months.^28^ Tarlatamab subsequently received FDA accelerated approval in May 2024 for the treatment of ES-SCLC with disease progression on or after platinum-based chemotherapy.

DeLLphi-301 did not have a comparator arm and no other clinical trials have evaluated tarlatamab in the 3L+ setting for SCLC. The phase 1 DeLLphi-300 study evaluated tarlatamab dosing and safety, although the median number of LOTs was 2.^29^ Ongoing studies assessing tarlatamab are mainly focused on early line treatment—these include the Phase 1 b DeLLphi-302 study of tarlatamab-immunotherapy combination in patients with ≥1 prior line, and the Phase 3 DeLLphi-304 study comparing tarlatamab vs chemotherapy in patients with 1L failure.^30^ Therefore, the relative efficacy of tarlatamab vs other 3L+ therapeutic options in this population is unknown. To address this evidence gap and inform treatment selection, we established an external control arm to facilitate an indirect treatment comparison (ITC) of the clinical outcomes between patients who received tarlatamab in DeLLphi-301 and those who received physician’s choice of therapies in a real-world setting, as recorded in the Flatiron Health Research database. A propensity score (PS)-weighting approach was used to adjust for differences in key prognostic factors between the two cohorts.

## Methods

### Data source

This study used patient-level data from the DeLLphi-301 trial and electronic health record (EHR)-derived de-identified data from the nationwide Flatiron Health Research database. The Flatiron Health database is a longitudinal, comprehensive oncology-focused real-world evidence resource that compiles de-identified EHRs from approximately 280 US cancer clinics (∼800 sites of care). The database includes over 4 million patient records, offering detailed insights into patient demographics, treatment patterns, outcomes, and healthcare utilization.^31^ Flatiron Health provided a customized real-world dataset of patients with SCLC that included data elements derived from structured data, unstructured data, and data curated from the EHRs by technology-enabled abstractors. The dataset included all data captured from January 2013 to October 2021 for eligible patients with ES-SCLC. This dataset was used to construct an external control arm of real-world physicians’ choice of therapies for SCLC in the 3L+ setting. The de-identified data were subject to obligations to prevent re-identification and protect patient confidentiality.

### Study design and population

The phase 2 DeLLphi-301 trial enrolled patients with SCLC who had received at least 2 prior lines of therapy, including platinum-based therapy. The design of the DeLLphi-301 trial has been reported previously by Ahn et al.^27^ Briefly, the study was performed in 3 parts: in part 1 (dose selection), patients were randomly assigned 1:1 to receive 28-day cycles of intravenous tarlatamab every 2 weeks at either a 10 mg or 100 mg dose until disease progression. In part 2 (dose expansion), patients were enrolled at the selected 10 mg dose until 100 patients had been enrolled from parts 1 and 2 combined, and in part 3, the safety of tarlatamab at the selected 10 mg dose was evaluated with a reduced duration of inpatient monitoring. The tarlatamab cohort of the present study included all patients who received 10 mg tarlatamab in DeLLphi-301 during parts 1 and 2, after excluding 2 patients who were enrolled despite not meeting the trial eligibility criterion of having ≥2 prior LOTs (*N* = 97). Included patients from DeLLphi-301 initiated tarlatamab between December 2021 and November 2022. Data collected for the patients from DeLLphi-301 covered December 2021 to October 2023.

**Figure 1.**
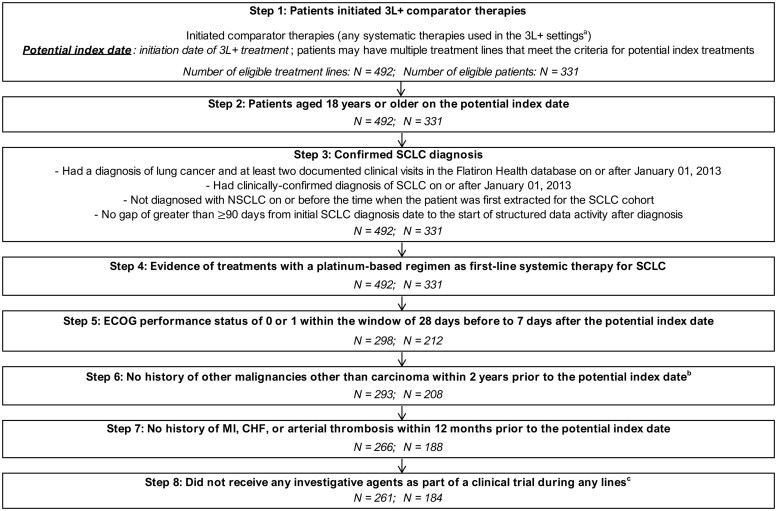
Sample Selection flowchart for the comparator therapies cohort. Abbreviations: 3L+, third line and beyond; CHF, congestive heart failure; ECOG PS, Eastern Cooperative Oncology Group Performance Status; MI, myocardial infarction; PD-1/L1, programmed death 1 or ligand 1; SCLC, small cell lung cancer. Notes: ^a^Any systematic therapies used in the 3L+ settings could include the following agents: cyclophosphamide/doxorubicin/vincristine (CAV), topotecan, irinotecan, lurbinectedin, platinum-based regimens with or without immunotherapy, immunotherapy only regimens, taxanes (paclitaxel and docetaxel), and others. ^b^Among the step 6 sample, 8 patients with 11 eligible treatment lines had carcinoma in situ of the breast, gastrointestinal tract, genitourinary system, gynecological organs, or lung. These patients were not excluded during sample selection to be consistent with DeLLphi-301 trial inclusion criteria. ^c^Among the step 8 sample, 15 patients with 20 eligible treatment lines had evidence of pneumonitis during the 90 days prior to the potential index date, and 9 patients with 10 eligible treatment lines had had brain or central nervous system (CNS) metastases prior to the potential index date and no evidence of treatment for brain or CNS metastases that was completed at least 2 weeks prior to the potential index date. They were not excluded in the sample selection assuming that patients who initiated 3L+ treatments had pneumonitis or brain or CNS metastases under control.

The Flatiron Health database included 331 patients with SCLC who initiated 3L+ treatment for SCLC. To construct a comparable patient population (the comparator therapies cohort), patients from the Flatiron Health database were selected based on key inclusion/exclusion criteria from DeLLphi-301 (Table S1). All patients were aged 18 years or older at the initiation of 3L+ treatment (ie, having ≥2 prior LOTs). Included patients initiated 3L+ treatment between March 2018 and October 2021, to ensure inclusion of a contemporary cohort of patients. Of 311 total patients, 119 (36%) who initiated 3L+ treatments were excluded due to Eastern Cooperative Oncology Group Performance Status (ECOG PS) of 2+ or unknown value. Additionally, 24 patients were excluded due to having other malignancies or comorbidity history and 4 patients were excluded as they received investigative agents as part of a clinical trial during any LOT. As a result, the comparator therapies cohort (*N* = 184) included patients who received comparator therapies for SCLC in 3L+ setting per DeLLphi-301 criteria (Figure 1).

Of note, 54 of the 184 patients in the comparator therapies cohort met the eligibility criteria at multiple LOTs. For these patients, one eligible LOT (defined as the index treatment) was identified using a PS-based approach. Detailed methodology on the index line selection is provided in the Supplementary Methods, and line distribution within the tarlatamab and Flatiron Health cohorts is summarized in Table S2.

### Study endpoints

The primary endpoint was OS, defined as the time from treatment initiation to the date of death due to any reason. Secondary endpoints included time to treatment discontinuation (TTD), defined as the time from treatment initiation to the treatment discontinuation or death, whichever occurred first, and time to next treatment or death (TTNTD), defined as the time from treatment initiation to the initiation of the next treatment or death, whichever occurred first. PFS and objective response rate (ORR) were considered as exploratory endpoints given the difference in criteria and cadence of assessments used to define response and progression in DeLLphi-301 (ie, RECIST 1.1) and Flatiron Health data (ie, documentation in various sources including radiographic imaging, pathology reports, and clinical examinations). Further details on the capture of these outcomes as well as censoring rules are included in Table S3.

### Statistical methods

#### Primary ITC analyses

To minimize potential confounding, a PS-weighting approach was used to adjust for differences in the distributions of key prognostic factors between the tarlatamab and comparator therapies cohorts before comparing efficacy outcomes. Identification of prognostic factors for adjustment in this ITC was based on a multi-step approach, incorporating a targeted literature review, empirical analyses per DeLLphi-301 data, and clinical inputs, and classified as being of high, medium, or low importance, based on clinical input (see Table S4 and Supplementary Methods). The chemotherapy-free interval (CFI) after 1L therapy was defined and imputed for 4 patients in the tarlatamab cohort with incomplete or missing data as described in the Supplementary Methods. There were no missing data on any of the other prognostic factors.

In the primary ITC analyses, patient characteristics at treatment initiation (ie, index date), including age, ECOG PS, disease stage at initial diagnosis, number of previous LOTs, CFI after 1L therapy, sex, presence of brain metastases, time from SCLC diagnosis to treatment initiation, and smoking status, were adjusted for based on standardized mortality ratio weights. Patients in the tarlatamab cohort were assigned a weight of 1, meaning their outcomes were unchanged. In contrast, patients in the comparator therapies cohort were weighted by PS/(1-PS), reflecting the outcomes of comparator therapies among the comparable DeLLphi-301 population. Standardized mean differences (SMD) between the tarlatamab and comparator therapy cohorts in patient characteristics determined to be prognostic factors were calculated before and after ­weighting to assess the balance between the two cohorts. Absolute SMD values of <0.25 were considered to reflect adequate balance on the adjusted confounders across the treatment cohorts after weighting.^32^^,^^33^

For time-to-event outcomes, hazard ratios (HRs) comparing tarlatamab versus comparator therapies were estimated before and after PS-weighting using Cox proportional hazards models. For ORR, odds ratios (ORs) were estimated before and after weighting using logistic regression models. The 95% CIs for HRs and ORs were estimated based on bootstrapping. To fully explain the estimated HRs and ORs between tarlatamab and comparator therapies with respect to potential unmeasured confounding, *E*-values were calculated for time-to-event outcomes to quantify robustness of the observed tarlatamab-outcome associations to potential unadjusted confounding.^34^  *E*-values quantify the minimum strength of association on the risk ratio scale that an unmeasured confounder would need to have with both the exposure and the outcome, conditional on already adjusted covariates, to fully explain away an observed exposure-outcome association. Large *E*-values, especially when analyses have already been adjusted for key confounding factors, indicate that the observed tarlatamab-outcome associations are unlikely to be fully explained away by unmeasured confounding.^35^

#### Sensitivity ITC analyses

Four sensitivity analyses were performed to assess the robustness of the results. In sensitivity analysis I, the analyses of PFS were repeated using only patients from the comparator therapies cohort whose progression was based on radiographic evidence, aligning more closely with the imaging-based evaluation in DeLLphi-301. In sensitivity analysis II, all analyses were repeated while also adjusting for prognostic factors considered to be of lower importance, including race, prior exposure to PD-1 and PD-L1 inhibitors, and presence of liver metastasis at index. In sensitivity analysis III, all analyses were repeated comparing tarlatamab vs a more restricted comparator cohort which excluded patients treated with lurbinectedin or PD-1/L1 inhibitor containing regimens to better reflect available therapies outside the US where regulatory approval or reimbursement of these newer treatments in 3L+ is likely to be limited. In sensitivity analysis IV, the tarlatamab cohort was compared with a further restricted contemporary comparator cohort of patients initiating 3L+ therapies on or after March 18, 2019, reflecting the therapeutic landscape for patients with ES-SCLC after atezolizumab + platinum-based chemotherapy became 1L standard of care. The same methodology and adjustment factors as main analysis used in the primary analyses were considered.

## Results

### Index treatment and line distribution

For the tarlatamab cohort (Table 1), the most common index LOT was 3L (67.0%), followed by 4L (18.6%), and further LOTs (5L: 7.2%, 6L: 6.2%, and 7L: 1.0%). For the comparator therapies cohort, the most common index LOT was also 3L (87.0%), followed by 5L (7.1%), and others (4L 4.3%, 6L 1.1%, and 7L 0.5%) after index LOT selection. The most common index treatment in the comparator therapies cohort was lurbinectedin (17.9%), followed by topotecan (15.2%), nivolumab (13.0%), paclitaxel (8.2%), and pembrolizumab (6.5%), nivolumab + ipilimumab (4.9%), carboplatin + irinotecan (4.3%), carboplatin + etoposide (3.8%), gemcitabine (3.3%), irinotecan (3.3%), and others.

**Table 1.** Line of therapy distribution for tarlatamab cohort and comparator therapies cohort and index line regimen distribution of the comparator therapies cohort after line selection.

Line distribution	Tarlatamab cohort (DeLLphi-301)	Comparator therapies cohort (Flatiron Health)	
	Number of patients (*N* = 97)	Number of patients (*N* = 184)	
**3L**	65 (67.0%)	160 (87.0%)	
**4 L**	18 (18.6%)	8 (4.3%)	
**5 L**	7 (7.2%)	13 (7.1%)	
**6 L**	6 (6.2%)	2 (1.1%)	
**7 L**	1 (1.0%)	1 (0.5%)	
**Regimen distribution in the comparator therapies (Flatiron Health) cohort, *N* (% of 184)**			
**Lurbinectedin**			33 (17.9%)
**Topotecan**			28 (15.2%)
**Nivolumab**			24 (13.0%)
**Paclitaxel**			15 (8.2%)
**Pembrolizumab**			12 (6.5%)
**Ipilimumab + nivolumab**			9 (4.9%)
**Carboplatin + irinotecan**			8 (4.3%)
**Carboplatin + etoposide**			7 (3.8%)
**Gemcitabine**			6 (3.3%)
**Irinotecan**			6 (3.3%)
**Cisplatin + irinotecan**			3 (1.6%)
**Docetaxel**			3 (1.6%)
**Others**			30 (16.3%)

Abbreviation: L, line.

### Baseline characteristics

The baseline characteristics for the tarlatamab and comparator therapies cohorts prior to and post-PS weighting are presented in Table 2. Before weighting, there were imbalances (absolute SMD > 0.25) between the two cohorts on age, sex, race/ethnicity, and number of previous LOTs. The tarlatamab cohort was slightly younger on average, had larger proportions of males and non-White patients, a lower proportion with 2 previous LOTs (ie, index LOT is 3L), and a higher proportion with 3 previous LOTs. After weighting, the two cohorts were well balanced across adjusted variables, reflected by all absolute SMD values <0.25. Among variables that were not adjusted for using PS-weighting, previous use of PD-1/PD-L1 inhibitors was relatively balanced, while some imbalance remained for race/ethnicity and liver metastasis. In sensitivity analysis II, previous use of PD-1/PD-L1 inhibitors, race/ethnicity, and liver metastasis were also weighted and achieved balance between the cohorts.

**Table 2.** Patient characteristics for tarlatamab and comparator therapies cohorts before and after PS weighting.

		Before weighting		After weighting	
Baseline characteristics	Tarlatamab (DeLLphi-301) (*N* = 97)	Comparator therapies (Flatiron Health) (*N* = 184)	SMD	Comparator therapies (Flatiron Health) (ESS = 77)	SMD
**Age at index (years), mean ± SD**	63.5 ± 8.7	65.6 ± 8.0	−0.255	63.8	−0.040
**Sex, *n* (%)**					
** Female**	26 (26.8%)	99 (53.8%)	−0.573	25.6%	0.028
** Male**	71 (73.2%)	85 (46.2%)	0.573	74.4%	−0.028
**Race/ethnicity, *n* (%)**					
** White**	55 (56.7%)	143 (77.7%)	−0.459	75.9%	−0.415
** Non-White**	42 (43.3%)	41 (22.3%)	0.459	24.1%	0.415
**Smoking history prior to index, *n* (%)**					
** Ever smoked**	89 (91.8%)	179 (97.3%)	−0.245	94.0%	−0.089
** Never smoked**	8 (8.2%)	5 (2.7%)	0.245	6.0%	0.089
**ECOG PS at index, *n* (%)**					
** 0**	25 (25.8%)	56 (30.4%)	−0.104	31.4%	−0.124
** 1**	72 (74.2%)	128 (69.6%)	0.104	68.6%	0.124
**Number of previous lines of therapies, *n* (%)**					
** 2**	65 (67.0%)	160 (87.0%)	−0.488	68.1%	−0.023
** 3**	18 (18.6%)	8 (4.3%)	0.458	17.0%	0.041
** >3**	14 (14.4%)	16 (8.7%)	0.180	14.9%	−0.014
**CFI after 1L therapy, *n* (%)^a^**					
** <90 days**	19 (19.6%)	46 (25.0%)	−0.130	17.1%	0.065
** ≥90 and <180 days**	41 (42.3%)	58 (31.5%)	0.224	39.5%	0.055
** ≥180 days**	37 (38.1%)	80 (43.5%)	−0.109	43.4%	−0.107
**Time from SCLC diagnosis to index (years), mean ± SD**	1.6 ± 0.8	1.4 ± 0.8	0.217	1.6	−0.032
**TNM stage at diagnosis, *n* (%)**					
** Stage I-II**	3 (3.1%)	12 (6.5%)	−0.161	4.5%	−0.073
** Stage III**	18 (18.6%)	35 (19.0%)	−0.012	16.5%	0.055
** Stage IV**	65 (67.0%)	116 (63.0%)	0.083	65.4%	0.033
** Unknown**	11 (11.3%)	21 (11.4%)	−0.002	13.6%	−0.068
**Previous use of PD-1/PD-L1 inhibitors, *n* (%)**	71 (73.2%)	117 (63.6%)	0.208	65.7%	0.164
**Brain metastases prior to or on index, *n* (%)**	22 (22.7%)	62 (33.7%)	−0.247	20.6%	0.051
**Liver metastases prior to or on index, *n* (%)**	37 (38.1%)	91 (49.5%)	−0.229	52.1%	−0.283

a For both the tarlatamab (DeLLphi-301) and comparator therapies (Flatiron Health) cohorts, CFI after 1L therapy was calculated as time interval between latest treatment end date across all platinum-based regimens within 1L to earliest treatment start date of 2L treatment. When only month and year were available for the end date of 1L platinum-based treatment, day was imputed as the last day of the month; when only month and year were available for the start date of 2L treatment, day was imputed as the first day of the month. Unknown CFI was imputed as ≥180 days.

Abbreviations: CFI, chemotherapy-free interval; ECOG PS, Eastern Cooperative Oncology Group Performance Status; ESS, effective sample size; L, line; PD-1, programmed cell death protein 1; PD-L1, programmed death ligand 1; PS, propensity score; SCLC, small cell lung cancer; SD, standard deviation; SMD, standardized mean difference; TNM, tumor, node, metastasis.

### Primary ITC analyses

#### Overall survival

**Figure 2.**
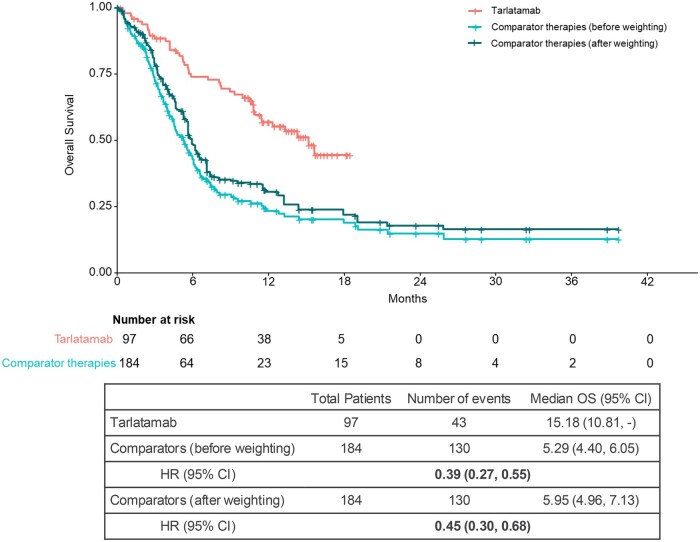
Comparison of OS between tarlatamab and comparator therapies cohort. Abbreviations: CI, confidence interval; HR, hazard ratio; OS, overall survival.

The median OS for the tarlatamab cohort was 15.2 months (95% CI, 10.8, NE) compared to 5.3 (4.4, 6.1) months before weighting and 6.0 (5.0, 7.1) months after weighting for the comparator therapies cohort. After weighting, greater OS benefit was observed in the tarlatamab cohort compared with the comparator therapies cohort (HR [95% CI], 0.45 [0.30, 0.68]) (Figure 2). The *E*-value for the observed association between tarlatamab and OS was 2.85. This indicates that a hypothetical unmeasured confounder would need to be strongly associated with both the treatment and OS with a risk ratio of 2.85 each, above and beyond the already adjusted high and medium priority confounders, to entirely explain away the HR of 0.45.

#### PFS and ORR

Four patients (2%) were excluded from the comparator therapies cohort for the PFS analysis because there was no progression assessment after the initiation of the index treatment (ie, the index date). The median PFS (95% CI) was 4.9 (2.9, 6.7) months for the tarlatamab cohort compared to 2.6 (2.2, 3.1) months before weighting, and 3.1 (2.3, 3.7) months after weighting for the comparator therapies cohort (HR [95% CI], 0.61 [0.43, 0.90]) (Figure S1). The *E*-value for the observed association between tarlatamab and PFS was 2.15. The tarlatamab cohort had significantly higher ORR (40%) compared with the comparator therapies cohort (18% before weighting and 19% after weighting), with an OR of 2.80 (95% CI, 1.44-5.83; *P* = .004) after weighting.

#### TTD and TTNTD

**Figure 3.**
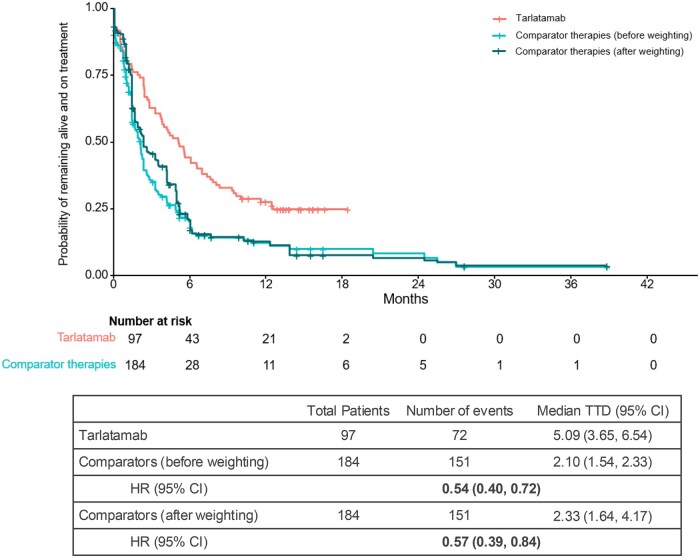
Comparison of TTD between tarlatamab and comparator therapies cohort. Abbreviations: CI, confidence interval; HR, hazard ratio; TTD, time to treatment discontinuation.

The median TTD for the tarlatamab cohort was 5.1 (95% CI, 3.7-6.5) months compared to 2.1 (1.5, 2.3) months before weighting and 2.3 (1.6, 4.2) months after weighting for the comparator therapies cohort (HR [95% CI], 0.57 [0.39, 0.84]) (Figure 3). The *E*-value for the observed association between tarlatamab and TTD was 2.31.

The median TTNTD was 10.2 (95% CI, 6.2-12.3) months for the tarlatamab cohort compared to 3.7 (3.2, 4.4) months before weighting and 4.3 (3.2, 5.7) months after weighting for the comparator therapies cohort (HR [95% CI], 0.45 [0.30, 0.66]) (Figure S2). The *E*-value for the observed association between tarlatamab and TTNTD was 2.86.

### Sensitivity analyses

The findings from the 4 sensitivity analyses were consistent with those of the primary analyses (Table 3).

**Table 3.** Summary of comparative results (HR or OR, 95% CI) between the tarlatamab and comparator therapies cohorts after weighting in the sensitivity analyses.

	Sensitivity analysis 1, with PFS defined based on radiographical evidence only	Sensitivity analysis 2, also adjusting for factors ranked of lower importance	Sensitivity analysis 3, with patients receiving 3L+ comparator therapies available outside of the US (ie, exclusion of lurbinectedin and PD-L1 inhibitors)			Sensitivity analysis 4, with patients receiving 3L+ comparator therapies on or after 03/18/2019 (same methodology and adjustment factors as main analysis)
			3a. Same methodology and adjustment factors as main analysis	3b. PFS based on radiographical evidence only (in the comparator cohort)	3c. Also adjusting for factors ranked of lower importance^a^	
**OS**	-	0.48 (0.31, 0.75)	0.45 (0.28, 0.74)	-	0.46 (0.29, 0.80)	0.48 (0.30, 0.76)
**TTD**	-	0.58 (0.41, 0.85)	0.47 (0.32, 0.70)	-	0.49 (0.32, 0.76)	0.57 (0.39, 0.83)
**TTNTD**	-	0.47 (0.32, 0.70)	0.43 (0.28, 0.68)	-	0.45 (0.28, 0.71)	0.44 (0.29, 0.66)
**PFS**	0.62 (0.43, 0.91)	0.63 (0.44, 0.92)	0.61 (0.41, 0.94)	0.61 (0.41, 0.95)	0.64 (0.42, 0.99)	0.60 (0.41, 0.86)
**ORR**	-	2.52 (1.23, 5.40)	2.29 (1.05, 5.58)	-	2.07 (0.82, 5.49)	2.64 (1.33, 6.08)

a Factors considered to be of lower clinical importance included previous use of PD-1 or PD-L1, liver metastases, and race.

Abbreviations: CI, confidence interval; HR, hazard ratio; L, line; OR, odds ratio; ORR, objective response rate; OS, overall survival; PFS, progression-free survival; TTNTD, time to next treatment or death; TTD, time to treatment discontinuation.

## Discussion

With the introduction of tarlatamab as a novel therapy for ES-SCLC in the relapsed disease setting, it is valuable to understand its relative effectiveness vs comparator therapies to guide patient access and treatment selection. In the absence of head-to-head trials, this study utilized patient-level data from the DeLLphi-301 clinical trial and the Flatiron Health database to indirectly compare the treatment effect, in terms of OS, PFS, TTD, TTNTD, and ORR, of tarlatamab and physicians’ choice of comparator therapies in 3L+ setting. The results of the primary analyses indicated that tarlatamab was associated with a significantly lower hazard of death, progression, treatment discontinuation, and initiation of subsequent treatment or death, as well as a higher response rate, than comparator therapies received by real-world patients with medical care recorded in the Flatiron Health network in the US.

The findings were consistent across all sensitivity analyses which were defined a priori, reinforcing the robustness of the study results and, to the extent feasible, addressing potential concerns regarding cohort differences between the tarlatamab and comparator therapies cohorts that could affect the interpretation of the relative treatment effects. In sensitivity analysis I, adjusting the PFS assessment method for the comparator therapies cohort (restricting to radiographic imaging only) to align more closely with the tarlatamab cohort resulted in HRs nearly identical to those of the primary analysis (0.62 vs 0.61). This suggests that the method of progression assessment does not meaningfully impact the observed associations. Sensitivity analysis II, which adjusted for all identified potential confounders—including those of lower importance—produced results consistent with the primary analysis, indicating the robustness of the estimated treatment effect against potential known and measurable confounding factors. In sensitivity analysis III, comparing tarlatamab to a subset of comparator therapies excluding lurbinectedin and PD-1/L1 regimens, showed similar results across all outcomes. These findings highlight the lack of a standard of care in 3L+ ES-SCLC, driven in part by the similarly poor effectiveness across available treatments in this heavily pre-treated population, and suggest that the ITC results are applicable across regions. Finally, the results of sensitivity analysis IV, comparing to tarlatamab to real-world 3L+ therapies in patients initiating treatment on or after March 18, 2019 (ie, FDA approval of 1L atezolizumab + platinum-based chemotherapy for ES-SCLC), were also similar to and support the robustness of the primary analysis. This analysis reflects recent trends in the treatment landscape for ES-SCLC, including the approval of 1L atezolizumab + platinum-based chemotherapy and withdrawal of nivolumab in 2020 after the confirmatory CheckMate-451 trial did not meet its primary endpoint (OS benefit).

The strength of this study is the use of high-quality, patient-level data to conduct an indirect treatment comparison. The DeLLphi-301 trial is the only clinical trial that assessed tarlatamab as 3L+ treatment for SCLC. Comparator therapies data were sourced from the Flatiron Health Research database, a well-established real-world data source known for its high-quality, longitudinal, patient-level, de-identified clinical information. With the recent FDA accelerated approval of tarlatamab as 2L+ treatment for ES-SCLC, as well as ongoing trials evaluating tarlatamab in 2L, future studies comparing tarlatamab with other therapy options in earlier treatment lines are warranted.

We implemented several best-practice measures to account for between-study differences between DeLLphi-301 and Flatiron Health data to the extent possible. With this approach, the DeLLphi-301 trial inclusion/exclusion criteria were applied step-by-step to the Flatiron Health data, enabling the selection of an external control arm that more closely resembles the trial population. Moreover, in the absence of randomization, careful adjustment for differences in confounding factors between the two cohorts is critical. This study took a rigorous approach to identify and adjust for a comprehensive list of confounders, minimizing bias to the extent possible. While bias due to unmeasured/unknown confounders cannot be entirely ruled out in a non-randomized study, the *E*-values (ranging from 2.15 to 2.86) suggests that any unmeasured confounders would have to be both highly imbalanced across groups and strongly associated with the outcomes, independent of the already adjusted-for prognostic factors, to explain away the observed associations. Thus, it is unlikely that these findings are explained away by residual or unmeasured confounding.

To our knowledge, this is the first study that has compared tarlatamab with comparator therapies in 3L+ setting for previously treated SCLC using patient-level data. A similar patient-level ITC conducted by Keeping et al. (2020) also used Flatiron Health data, applying nivolumab trial criteria to form an external control arm for 3L SCLC.^36^ The most-used comparator treatment was paclitaxel and topotecan in Keeping et al., while it was lurbinectedin in our study. This may be explained by the earlier timeframe of Flatiron Health data in Keeping et al. (January 2011 to September 2017) preceding lurbinectedin approval (June 2020),^37^ while the data used in this analysis encompass it (January 2013 to October 2021). Keeping et al. reported a lower median OS for comparator therapies of 3.8 months before weighting, which remained similar after weighting, than the 5.3 months pre-weighting and 6.0 months post-weighting observed in this study. This difference could be related to their exclusion of patients with any prior immunotherapy use and inability to confirm ECOG 0–1 at 3L initiation due to lack of availability.

The findings of this study should be considered in light of several limitations. First, the comparator therapies cohort used data abstracted from real-world EHRs, which may introduce measurement error via mistakes or omissions in data entry into the underlying EHR. While some errors in the underlying EHR data may be present, measurement error and misclassification due to errors in abstraction from the EHR are likely to be low given the use of trained abstractors and systematic, modular abstraction guidelines, and quality checks implemented by Flatiron Health. Outcome assessment for PFS and ORR in Flatiron Health may also be subject to measurement error given the differences in criteria for progression and response ascertained across trial and real-world settings. Potential measurement error with regards to the cadence of radiographic scans in a trial vs a real-world setting could not be fully addressed by the sensitivity analysis and remains a plausible limitation. Second, while the inclusion/exclusion criteria from DeLLphi-301 were applied to the comparator therapies cohort to the extent possible, some criteria such as measurable lesions as per RECIST 1.1 within 21 days before the treatment initiation could not be applied due to the lack of data in similar format or RECIST-based assessments in the Flatiron Health database. This introduces the possibility of selection bias which cannot be completely ruled out, if patients who were excluded due to missing data had different outcomes than those who were included. Third, we attempted to minimize confounding by adjusting for a comprehensive list of variables using PS methods. Nevertheless, some residual confounding may remain, although it is unlikely to materially affect the results. As borne out by the *E*-value calculations, any unmeasured confounders would have to both be highly imbalanced across groups and strongly associated with outcomes independent of the already adjusted high and medium priority confounding factors to explain away associations of the magnitude observed here. Considering these points, it is unlikely that these findings are explained by residual or unmeasured confounding. Lastly, there existed differences in the time frames of data collection between DeLLphi-301 (December 2021 to October 2023) and Flatiron Health data (January 2013 to October 2021), which could potentially lead to bias in the historical comparisons if there were changes in clinical management. However, the index date in the comparator therapies cohort from Flatiron Health database ranged from 2018 to 2021 to reflect a more contemporaneous population with the tarlatamab cohort from DeLLphi-301. Additionally, no major differences were expected over these time frames given the lack of advances in care of 3L+ SCLC during that time.

## Conclusions

The results of this ITC indicate that, relative to comparator therapies, tarlatamab was consistently associated with significantly longer OS and PFS, higher ORR, and longer TTD and TTNTD across all analyses. These results strongly suggest that tarlatamab may confer a clinically meaningful benefit for patients with SCLC who have received at least 2 prior lines of therapies, and that tarlatamab is an encouraging option for improving clinical outcomes in this population.

## Supplementary Material

oyaf256_Supplementary_Data

## Data Availability

The data that support the findings of this study originated from and are the property of Flatiron Health, Inc., which has restrictions prohibiting the authors from making the data set publicly available. Requests for data sharing by license or by permission for the specific purpose of replicating results in this manuscript can be submitted to PublicationsDataAccess@flatiron.com.

## References

[1] Rudin CM , Brambilla E, Faivre-Finn C, Sage J. Small-cell lung cancer. Nat Rev Dis Primers. 2021;7:3. 33446664 10.1038/s41572-020-00235-0 PMC8177722

[2] American Cancer Society. Key statistics for lung cancer. Accessed November 11, 2024, https://www.cancer.org/cancer/types/lung-cancer/about/key-statistics.html

[3] McNamee N , da Silva IP, Nagrial A, Gao B. Small-cell lung cancer-an update on targeted and immunotherapies. Int J Mol Sci. 2023;24:8129. 37175833 10.3390/ijms24098129 PMC10179261

[4] Farago AF , Keane FK. Current standards for clinical management of small cell lung cancer. Transl Lung Cancer Res. 2018;7:69-79. 29535913 10.21037/tlcr.2018.01.16 PMC5835595

[5] Petty WJ , Paz-Ares L. Emerging strategies for the treatment of small cell lung cancer: a review. JAMA Oncol. 2023;9:419-429. 36520421 10.1001/jamaoncol.2022.5631

[6] Shaw J , Pundole X, Balasubramanian A, et al Recent treatment patterns and real-world survival following first-line anti-PD-L1 treatment for extensive-stage small cell lung cancer. Oncologist. 2024;29:1079-1089. 39349396 10.1093/oncolo/oyae234 PMC11630770

[7] Dingemans AC , Früh M, Ardizzoni A, et al Small-cell lung cancer: ESMO clinical practice guidelines for diagnosis, treatment and follow-up^⋆^. Ann Oncol. 2021;32:839-853. 33864941 10.1016/j.annonc.2021.03.207 PMC9464246

[8] National Institute for Health and Care Excellence. Lung cancer: diagnosis and management. Accessed November 10, 2024, https://www.nice.org.uk/guidance/ng122 31211540

[9] National Comprehensive Cancer Network (NCCN). NCCN clinical practice guidelines in oncology (NCCN Guidelines^®^): small cell lung cancer. Version 3.2024. June 11, 2024.

[10] Igawa S , Yamamoto N, Ueda S, et al Evaluation of the recommended dose and efficacy of amrubicin as second- and third-line chemotherapy for small cell lung cancer. J Thorac Oncol. 2007;2:741-744. 17762341 10.1097/JTO.0b013e31811f46f0

[11] Xu Y , Huang Z, Lu H, et al Apatinib in patients with extensive-stage small-cell lung cancer after second-line or third-line chemotherapy: a phase II, single-arm, multicentre, prospective study. Br J Cancer. 2019;121:640-646. 31523058 10.1038/s41416-019-0583-6 PMC6889407

[12] Ready N , Farago AF, de Braud F, et al Third-line nivolumab monotherapy in recurrent SCLC: CheckMate 032. J Thorac Oncol. 2019;14:237-244. 30316010 10.1016/j.jtho.2018.10.003 PMC8050700

[13] Cheng Y , Wang Q, Li K, et al Anlotinib vs placebo as third- or further-line treatment for patients with small cell lung cancer: a randomised, double-blind, placebo-controlled phase 2 study. Br J Cancer. 2021;125:366-371. 34006926 10.1038/s41416-021-01356-3 PMC8329046

[14] Wu D , Nie J, Hu W, et al A phase II study of anlotinib in 45 patients with relapsed small cell lung cancer. Int J Cancer. 2020;147:3453-3460. 32557583 10.1002/ijc.33161 PMC7689882

[15] He Z , Zhou H, Wang J, et al Apatinib with etoposide capsules as a third- or further-line therapy for extensive-stage small cell lung cancer: an open-label, multicenter, single-arm phase II trial. Transl Lung Cancer Res. 2021;10:889-899. 33718030 10.21037/tlcr-20-1235 PMC7947412

[16] Udagawa H , Akamatsu H, Tanaka K, et al Phase I safety and pharmacokinetics study of rovalpituzumab tesirine in Japanese patients with advanced, recurrent small cell lung cancer. Lung Cancer. 2019;135:145-150. 31446987 10.1016/j.lungcan.2019.07.025

[17] Sun JM , Kim JR, Do IG, et al A phase-1b study of everolimus plus paclitaxel in patients with small-cell lung cancer. Br J Cancer. 2013;109:1482-1487. 23963141 10.1038/bjc.2013.467 PMC3776982

[18] Tarhini A , Kotsakis A, Gooding W, et al Phase II study of everolimus (RAD001) in previously treated small cell lung cancer. Clin Cancer Res. 2010;16:5900-5907. 21045083 10.1158/1078-0432.CCR-10-0802

[19] Carbone DP , Morgensztern D, Le Moulec S, et al Efficacy and safety of rovalpituzumab tesirine in patients with DLL3-expressing, ≥ 3rd line small cell lung cancer: results from the phase 2 TRINITY study. Pneumologie. 2019;73:P320. 10.1158/1078-0432.CCR-19-1133 PMC7105795 31506387

[20] Cheng Y , Wang Q, Li K, et al P2.12-26 the impact of anlotinib for relapsed SCLC patients with brain metastases: a subgroup analysis of ALTER 1202. J Thorac Oncol. 2019;14:S823-S824.

[21] Chung HC , Piha-Paul SA, Lopez-Martin J, et al Pembrolizumab after two or more lines of previous therapy in patients with recurrent or metastatic SCLC: results from the KEYNOTE-028 and KEYNOTE-158 studies. J Thorac Oncol. 2020;15:618-627. 31870883 10.1016/j.jtho.2019.12.109

[22] Ott PA , Elez E, Hiret S, et al Pembrolizumab in patients with extensive-stage small-cell lung cancer: results from the phase Ib KEYNOTE-028 study. J Clin Oncol. 2017;35:3823-3829. 28813164 10.1200/JCO.2017.72.5069

[23] Gray JE , Heist RS, Starodub AN, et al Therapy of small cell lung cancer (SCLC) with a topoisomerase-i-inhibiting antibody-drug conjugate (ADC) targeting trop-2, sacituzumab govitecan. Clin Cancer Res. 2017;23:5711-5719. 28679770 10.1158/1078-0432.CCR-17-0933

[24] Morgensztern D , Besse B, Greillier L, et al Efficacy and safety of rovalpituzumab tesirine in third-line and beyond patients with DLL3-expressing, relapsed/refractory small-cell lung cancer: results from the phase II TRINITY study. Clin Cancer Res. 2019;25:6958-6966. 31506387 10.1158/1078-0432.CCR-19-1133 PMC7105795

[25] United States Food and Drug Administration. Highlights of prescribing information: IMDELLTRA (tarlatamab-dlle). Accessed June 5, 2024, https://www.accessdata.fda.gov/drugsatfda_docs/label/2024/761344s000lbl.pdf

[26] ClinicalTrials.gov. A phase 2 study of tarlatamab in patients with small cell lung cancer (SCLC) (DeLLphi-30). Accessed November 12, 2024, https://clinicaltrials.gov/study/NCT05060016

[27] Ahn M-J , Cho BC, Felip E, et al Tarlatamab for patients with previously treated small-cell lung cancer. N Engl J Med. 2023;389:2063-2075. 37861218 10.1056/NEJMoa2307980

[28] Sands J , Cho BC, Ahn MJ, et al OA10.03 tarlatamab sustained clinical benefit and safety in previously treated SCLC: DeLLphi-301 phase 2 extended follow-up. J Thorac Oncol. 2024;19:S30-S31.

[29] Paz-Ares L , Champiat S, Lai WV, et al Tarlatamab, a first-in-class DLL3-targeted bispecific t-cell engager, in recurrent small-cell lung cancer: an open-label, phase i study. J Clin Oncol. 2023;41:2893-2903. 36689692 10.1200/JCO.22.02823 PMC10414718

[30] Rudin CM , Mountzios GS, Sun L, et al Tarlatamab versus chemotherapy (CTx) as second-line (2L) treatment for small cell lung cancer (SCLC): primary analysis of Ph3 DeLLphi-304. J Clinc Oncol. 2025;43:LBA8008-LBA8008.

[31] Flatiron Health. Accessed November 12, 2024, https://flatiron.com/real-world-evidence/real-world-data

[32] Stuart EA , Lee BK, Leacy FP. Prognostic score-based balance measures can be a useful diagnostic for propensity score methods in comparative effectiveness research. J Clin Epidemiol. 2013;66:S84-S90.e1. 23849158 10.1016/j.jclinepi.2013.01.013 PMC3713509

[33] Rubin DB. Using propensity scores to help design observational studies: application to the tobacco litigation. Health Serv Outcomes Res Methodol. 2001;2:169-188.

[34] Mathur MB , Ding P, Riddell CA, VanderWeele TJ. Web site and R package for computing E-values. Epidemiology. 2018;29:e45-e47. 29912013 10.1097/EDE.0000000000000864 PMC6066405

[35] VanderWeele TJ , Ding P. Sensitivity analysis in observational research: introducing the e-value. Ann Intern Med. 2017;167:268-274. 28693043 10.7326/M16-2607

[36] Keeping ST , Cope S, Chan K, et al Comparative effectiveness of nivolumab versus standard of care for third-line patients with small-cell lung cancer. J Comp Eff Res. 2020;9:1275-1284. 33140652 10.2217/cer-2020-0134

[37] United States Food and Drug Administration. Highlights of prescribing information: ZEPZELCATM (lurbinectedin). Accessed November 13, 2024, https://www.accessdata.fda.gov/drugsatfda_docs/label/2020/213702s000lbl.pdf

