# Supplementary material for: Efficacy outcomes between tarlatamab and real-world physicians’ choice of therapies for previously treated extensive stage small cell lung cancer
Source: Oncologist. 2025 Aug 21;30(9):oyaf256. doi: 10.1093/oncolo/oyaf256 (PMC12459094; doi:10.1093/oncolo/oyaf256)
Supplement: oyaf256_Supplementary_Data [file oyaf256_supplementary_data.docx]

**SUPPLEMENTARY MATERIAL**

**Table S1. Summary of inclusion and exclusion criteria in tarlatamab and comparator therapies cohorts**

| Tarlatamab (DeLLphi-301) | Comparator therapies (Flatiron Health) |
| --- | --- |
| **Inclusion criteria** | |
| - Initiated tarlatamab 10 mg | - Initiated comparator therapies (i.e., any systematic therapies used in the 3L+ settings, including cyclophosphamide/doxorubicin/vincristine [CAV], topotecan, irinotecan, lurbinectedin, platinum-based regimens with or without immunotherapy, immunotherapy only regimens, taxanes [paclitaxel and docetaxel], and others) |
| - Male or female age 18 years or older at screening | - Male or female age 18 years or older at initiation of 3L+ treatment *(index date)* |
| - Histologically or cytologically confirmed SCLC | - Diagnosis of lung cancer (ICD-9 162.x or ICD-10 C34x, or C39.9) - Pathology consistent with SCLC (i.e., based on biopsy findings and/or that the provider explicitly documents a SCLC diagnosis.); not diagnosed with NSCLC on or before the time a patient was first extracted for the SCLC cohort |
| - Previously treated SCLC; progressed or recurred following 1 platinum-based regimen and at least 1 other prior LOT prior to tarlatamab initiation (i.e., patients are 3L+ at tarlatamab initiation) | - Evidence of treatment with a platinum-based regimen as front-line systemic (non-maintenance) therapy for SCLC, and ≥1 immediate subsequent (non-maintenance) line of therapy for SCLC after 1L treatment - Initiated a 3L+ systemic treatment - No gap of greater than ≥ 90 days from initial SCLC diagnosis date to the start of structured data activity after diagnosis |
| - ECOG PS of 0 or 1 at screening | - ECOG PS of 0 or 1 within a window of 28 days before or 7 days after initiation of 3L+ treatment |
| - No brain metastases at screening, or - Treated brain metastases, if definitive therapy was completed at least 2 weeks prior to first dose of tarlatamab, no evidence of CNS progression or only pseudoprogression at time of screening, and asymptomatic | - No brain metastases prior to initiation of 3L+ therapy, or - Patients with brain metastases who initiated 3L+ treatment. Initiating 3L+ treatment was considered to reflect that active management was in place or a justification for not actively treating brain metastases was made prior to initiating anti-cancer treatment. |
| - Adequate organ function, defined based on laboratory/test values for markers of hematological, coagulation, renal, hepatic, pulmonary and cardiac function | - Proxied by patients initiating 3L+ treatment, given that adequate organ function is required before initiating any anti-cancer therapies in clinical practice |
| **Exclusion criteria** | |
| - Untreated or symptomatic CNS metastases or leptomeningeal disease | - Untreated brain or CNS metastases as proxied by neither a record of treatment for brain metastases nor initiation of 3L+ SCLC treatment |
| - Evidence of interstitial lung disease or active, non-infectious pneumonitis | - Not applied to the Flatiron Health cohort. Initiating 3L+ treatment was considered to reflect that active management was in place or a justification for not actively treating interstitial lung disease or active non-infectious pneumonitis was made prior to initiating anti-cancer treatment. |
| - History of other malignancies within the past 2 years (with exceptions) | - Evidence of other malignancies except carcinoma in situ within 2 years prior to 3L+ treatment initiation |
| - History of MI, symptomatic CHF, or arterial thrombosis within 12 months of first dose of tarlatamab | - History of MI, CHF, or arterial thrombosis within 12 months of 3L+ treatment initiation |
| - Evidence of hepatitis B | - Not applied to the Flatiron Health cohort as this was not considered prognostic for outcomes in this population |
| - Evidence of hepatitis C | - Not applied to the Flatiron Health cohort as this was not considered prognostic for outcomes in this population |
| - Diagnosis of immunodeficiency (e.g., HIV/AIDS) or receiving systemic steroids or immunosuppressive therapy within 7 days prior to first dose of tarlatamab | - Not applied to the Flatiron Health cohort as this was not considered prognostic for outcomes in this population |
| - Prior therapy with tarlatamab | - Not applicable |
| - Prior anti-cancer therapy within 28 days prior to first dose of tarlatamab (except conventional chemotherapy or radiotherapy) | - Not applied to the Flatiron Health cohort as this was not considered as an important prognostic factor for outcomes in this population. |
| - Currently receiving or recently ended treatment in another investigational device or drug study | - Received any investigative agents as part of a clinical trial during any LOT (including 3L+) |

**Abbreviations:** 1L, first line; 3L+, third-line and beyond; AIDS, acquired immunodeficiency syndrome; CAV, cyclophosphamide/doxorubicin/vincristine; CHF, congestive heart failure; CNS, central nervous system; ECOG PS, Eastern Cooperative Oncology Group Performance Status; HIV, human immunodeficiency virus; ICD-9/10, International Classification of Disease- 9^th^/10^th^ edition; LOT, line of therapy; MI, myocardial infarction; NSCLC, non-small cell lung cancer; SCLC, small cell lung cancer.

**Table S2. Line distribution of tarlatamab cohort and comparator therapies before line selection**

|  | Tarlatamab cohort (DeLLphi-301) | Comparator therapies cohort (Flatiron Health) |
| --- | --- | --- |
|  |  | Before line selection |
|  | Number of patients (N=97) | Number of patients (N=184) |
| Number of patients with unique line | 97 (100%) | 130 (70.7%) |
| Number of patients with multiple lines |  |  |
| 2 potential index lines | 0 | 36 (19.6%) |
| 3 potential index lines | 0 | 14 (7.6%) |
| 4 potential index lines | 0 | 3 (1.6%) |
| 5 potential index lines | 0 | 1 (0.5%) |
| Line distribution | Number of lines (N=97) | Number of lines (N=261) |
| 3L | 65 (67.0%) | 177 (67.8%) |
| 4L | 18 (18.6%) | 58 (22.2%) |
| 5L | 7 (7.2%) | 21 (8.0%) |
| 6L | 6 (6.2%) | 4 (1.5%) |
| 7L | 1 (1.0%) | 1 (0.5%) |

**Abbreviations:** L, line

**Table S3. List of outcomes and definitions**

| Outcome | Tarlatamab (DeLLphi-301) | Comparator therapies (Flatiron Health) |
| --- | --- | --- |
| Primary outcome | | |
| OS | Defined as time from index date to death. Patients who were not known to have died by the end of the data cutoff date were censored at the date of last contact when they were known to be alive or by the end of data cutoff date, whichever was earlier.  Vital status and date of death if deceased were known through scheduled study visits and monitoring during the treatment period of the trial. Vital status and date of death if deceased during long-term follow-after the end of tarlatamab treatment was assessed through a mixture of clinic visits, telephone follow-up or chart review. | Defined as time from index date to death. Patients not known to have died by the end of the data cutoff date were censored at their last confirmed clinical activity date in the database.  Vital status and date of death if deceased were identified by combining mortality information from the structured and unstructured Flatiron Health EHR data, and publicly available Social Security Death Index data and obituary data (from obituaries, funeral homes and other sources). |
| Secondary outcomes | | |
| TTD | Defined as time from index date to treatment discontinuation or death, whichever occurred first. Patients who remained alive and on treatment were censored at their last recorded date of tarlatamab use | Defined as time from index date to treatment discontinuation or death, whichever occurred first. Patients were considered to have discontinued the index treatment if there was a gap of > 120 days after the last recorded date of index treatment, or if they initiated a subsequent line of treatment. Patients who remained alive and on the index treatment were censored at their last recorded date of index treatment use. |
| TTNTD | Defined as time from index date to initiation of the next treatment or death, whichever occurred first. Patients who remained alive and did not initiate a subsequent anti-cancer treatment were censored at their last recorded study visit. | Defined as time from index date to initiation of the next treatment or death, whichever occurred first. Patients who remained alive and did not have a subsequent LOT were censored at their last confirmed clinical activity date in the database. |
| Exploratory outcomes | | |
| PFS | Defined as time from index date to disease progression or death, whichever occurred first. Disease progression was assessed by blinded independent central review according to RECIST 1.1 criteria.  Censoring rules   - Patients who did not have disease progression or death and no subsequent anti-cancer treatment were censored at their last post-baseline disease assessment date. - Patients who did not disease progression or death who had subsequent anticancer treatment were censored at their last visit prior to initiating the new treatment - Disease progression or death that occurred after the initiation of the next LOT was not considered as an event; for those scenarios, patients were censored at the last disease assessment date prior to the initiation of the next LOT - Disease progression or death that occurred more than 14 weeks after the last disease assessment visit was not considered as an event; in these scenarios, patients were censored at the last disease assessment visit prior to progression or death. | Defined as time from index date to disease progression or death, whichever occurred first. Disease progression was retrospectively captured and identified based on clinical documentation of progression from different sources of evidence, which may have included radiographic imaging, pathology reports, or clinical examination.  Censoring rules   - Patients who did not have disease progression or death were censored at their last clinical note date. - Patients who did not have disease progression or death but did have subsequent anticancer treatment were censored at the earlier of their last clinic note date or the day before initiating the new treatment - Disease progression or death that occurred after the initiation of the next LOT was not considered as an event; for those scenarios, patients were censored at the earlier of their last clinic note date or the day before initiating the new treatment. - Disease progression or death occurred more than 14 weeks after the last disease assessment visit were not considered as an event; in these scenarios, patients were censored at the last disease assessment visit prior to progression or death. - Only progression events that occurred after 14 days after the index date were utilized in this analysis. - Patients with last clinic note date before the index date were excluded from the PFS analysis |
| ORR | Defined as the proportion of patients with CR or PR per RECIST v1.1 before initiation of a subsequent anti-cancer treatment, if any. Response assessments occurring after the start of the first subsequent anti-cancer therapy were not used to evaluate ORR. | Defined as the proportion of all treated patients with CR or PR. Response assessments were identified based on clinical documentation of progression from various sources of evidence, including interpretation of a radiology or pathology report, or clinical examination. CR or PR that occurred after the initiation of next LOT were not considered as an objective response associated with the index treatment. Patients without a response assessment record were considered as non-responders. |

**Abbreviations:** CR, complete response; EHR, electronic health records; LOT, line of therapy; ORR, objective response rate; OS, overall survival; PR, partial response; PFS, progression-free survival; PR, partial response; TTNTD, time to next treatment or death; TTD, time to treatment discontinuation.

**Table S4. Description of baseline adjustment factors in this study**

|  |  | **Definition/assessment** | |
| --- | --- | --- | --- |
| **Variable** | **Levels** | **Tarlatamab (DeLLphi-301)** | **Comparator therapies (Flatiron Health)** |
| **High importance** | | | |
| Age at index | Continuous variable | Derived based on date of birth and index date | Derived based on year of birth and index date |
| ECOG PS at index | 0, 1 | Assessed as part of the clinical evaluation on the day of tarlatamab administration | Assessed based on ECOG PS and corresponding dates recorded in the structured data in EHR |
| TNM disease stage at diagnosis | Stage I-II, stage III, stage IV, Unknown | Assessed based on medical history eCRF dated back to the original SCLC diagnosis | Assessed based on American Joint Committee on Cancer stage explicitly documented in EHR |
| Number of previous LOTs at index | 2 lines, 3 lines, and 3+ lines | Derived from treatment history captured in eCRF | Derived from treatment history captured in the Flatiron Health database |
| **Medium importance** | | | |
| CFI after 1L therapy* | <90 days, ≥ 90 and < 180 days, and ≥ 180 days | Time interval between latest treatment administration date across all platinum drugs within 1L to the initiation of 2L treatment | Time interval between latest drug episode date across all platinum drugs within 1L to the initiation of 2L treatment |
| Sex | Male, female | Assessed at baseline | Assessed at baseline |
| Presence of brain metastasis at index | Yes, No | Assessed based on MRI at trial screening | Assessed based on unstructured data in EHR of clinical or pathologic statement confirming metastatic site |
| Smoking status | Ever smoked, never smoked | Assessed at trial screening | Assessed based on unstructured data in EHR |
| Time from SCLC diagnosis to index | Continuous variable | Derived based on SCLC diagnosis date and index date | Derived based on SCLC diagnosis date and index date |
| **Lower importance** | | | |
| Race | White, non-White | Assessed at baseline | Assessed at baseline |
| Prior exposure of PD-1/PD-L1 inhibitors | Yes, No | Derived from treatment history captured in eCRF | Derived from treatment history captured in the Flatiron Health database |
| Presence of liver metastasis at index | Yes, No | Assessed based on MRI at trial screening | Assessed based on unstructured data in EHR of clinical or pathologic statement confirming metastatic site |

**Abbreviations:** CFI, chemotherapy-free interval; ECOG PS, Eastern Cooperative Oncology Group Performance Status; eCRF, electronic case report form; EHR, electronic health record; MRI, magnetic resonance imaging; PD-1, programmed cell death protein 1; PD-L1, programmed death ligand 1; SCLC, small cell lung cancer.


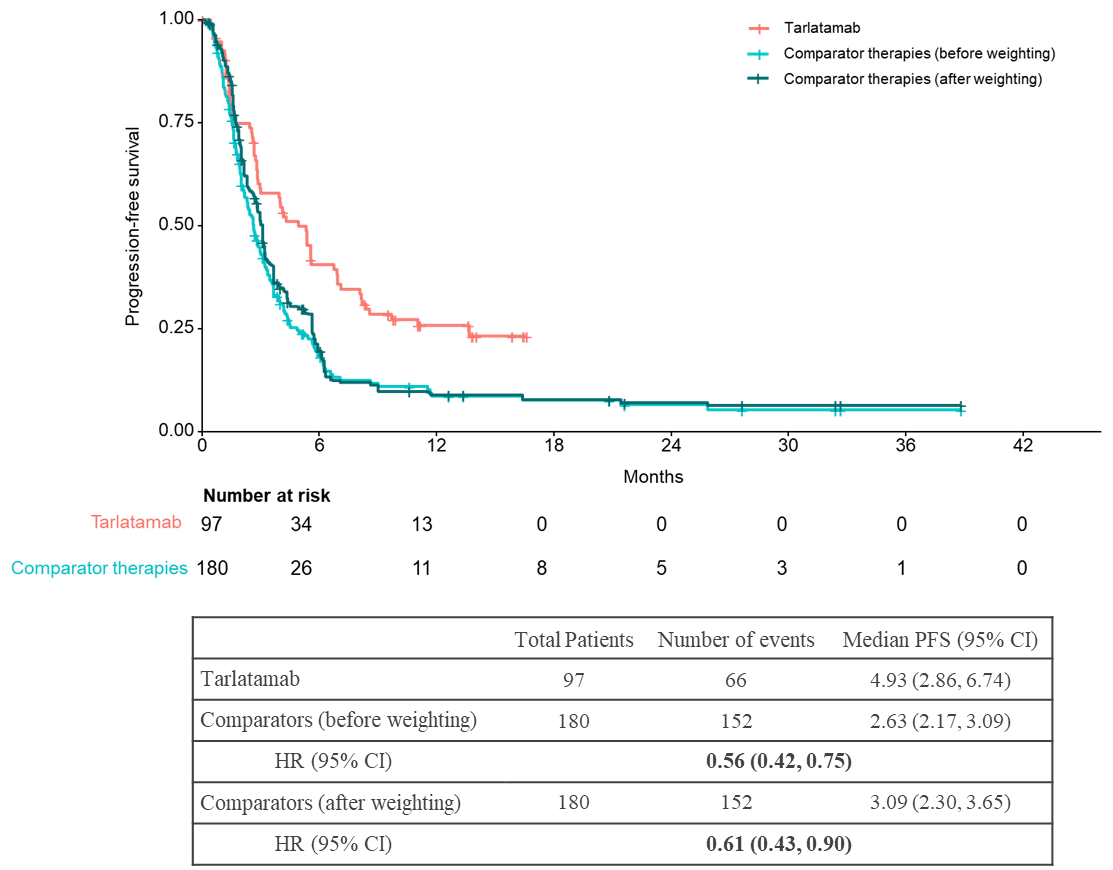
**Figure S1. Comparison of PFS between tarlatamab and comparator therapies cohort**

**Abbreviations:** CI, confidence interval; HR, hazard ratio; PFS, progression-free survival.

**Figure S2. Comparison of TTNTD between tarlatamab and comparator therapies cohort**


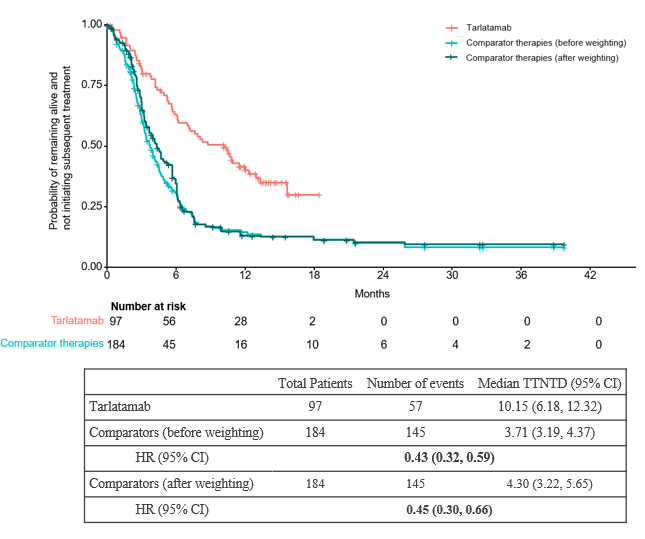


**Abbreviations:** CI, confidence interval; HR, hazard ratio; TTNTD, time to next treatment or death.

**Supplementary Methods**

***Index line selection***

A generalized estimating equation with logit link was used to estimate the propensity score per-patient per-line to be treated in DeLLphi-301 based on the prognostic factors deemed as high and medium importance, including age at index, sex, Eastern Cooperative Oncology Group performance status (ECOG PS) at index, Tumor/Node/Metastasis disease stage at diagnosis per American Joint Committee on Cancer staging, number of previous lines of therapy (LOTs) at index, chemotherapy-free interval (CFI) after first-line (1L) therapy, presence of brain metastases at index, time from small cell lung cancer (SCLC) diagnosis to index date, and smoking status (**Table S4**). The therapy line with the highest propensity score was selected as the index treatment.

***Identification of prognostic factors and potential treatment effect modifiers***

A multi-step approach, incorporating a targeted literature review, empirical analyses of available data, and expert input, was used to identify important prognostic factors in SCLC. In step 1, a targeted literature review identified two matching-adjusted indirect comparison (MAIC) studies in SCLC, Keeping et al. and Havensakul et al.^35,37^ The prognostic factors identified from these studies were included in a questionnaire for oncologists and clinical experts to rank the importance and prognostic effect of these factors in the context of deriving treatment benefit for patients with SCLC in the 3L setting. In the questionnaire, ECOG PS was unanimously considered the most important factor to predict prognosis, while other factors indicated to be very important were disease stage (limited vs extensive stage) and response to previous treatment (platinum sensitivity).

In step 2, univariate and multivariate Cox regressions were performed on the individual patient data (IPD) from DeLLphi-301 to screen the prognostic factors for statistical significance (p £0.05). The analyses indicated that two covariates are significant predictors of overall survival (OS): ECOG PS and presence of brain metastases, both confirmed by clinical opinion.

In step 3, meta-regression analysis was performed to explore the potential relationship between population characteristics and study outcomes from systematic literature reviews (SLRs) of 2L and 3L+ clinical studies in patients with relapsed/refractory SCLC utilizing all reported evidence on specified variables independent of timepoint of measurement. The meta-regression performed using extracted data identified five variables found to be statistically significant for at least one outcome (OS, PFS or ORR) at p <0.05. Patient characteristics predictive of outcomes included sex and treatment class. Other potential predictors of outcomes include ECOG PS, LOT, and extensive stage (ES) disease, with age showing no evidence of impact by the crude measure used (i.e., median/mean).

In step 4, the results from the prior three steps were submitted for comments by clinical experts for finalization of the list of potential adjustment factors considered for ITC. Based on the final clinical inputs, candidate adjustment factors were prioritized as follows:

- **Highest importance**: ECOG PS, age, disease stage, and number of previous LOTs
- **Medium importance:** CFI, sex, brain metastases, time from SCLC diagnosis
- **Lower importance:** previous use of PD-1 or PD-L1 inhibitors, liver metastases, race/ethnicity

***Definition and imputation of CFI***

For both the tarlatamab and comparator therapy cohorts, CFI after 1L therapy was calculated as the time interval between the latest recorded treatment end date across all platinum-based regimens within 1L to the earliest recorded treatment start date of 2L treatment. When only month and year were available for the end date of 1L platinum-based treatment, day was imputed as the last day of the month; when only month and year were available for the start date of second line (2L) treatment, day was imputed as the first day of the month. Four patients in the tarlatamab cohort with unknown CFI were imputed as having a CFI of ≥180 days; there was no missing data on CFI in the comparator therapy cohort.
